# Nervous Force. Its Origin and Physiology

**Published:** 1882-12

**Authors:** W. C. Barrett

**Affiliations:** Buffalo, N. Y.


AMERICAN JOURNAL

OF

DENTAL SCIENCE.

Vol. XVI. THIRD SERIES—DECEMBER, 1882. No. 8

### ARTICLE I.

### *Nervous Force: Its Origin and Physiology.*

BY W. C. BARRETT, M. D., D. D. S., BUFFALO, N. Y.

[Read before the American Dental Association at its Twenty-Second Annual Meeting, held in Cincinnati, August 1–5, 1882.]

There are two things of which I wish to speak, and these two things make up the sum of the whole universe so far as man can know: they are matter and force. I say the study of these must form the whole, the entirety of human research, so far as any positive knowledge or the hope of definite information is concerned. We may speculate and theorize and dogmatize upon things spiritual and metaphysical as much as we like, but concerning them we can by no possibility arrive at any definite conclusion, nor can we *prove* any assertion, it matters little how wild it may be, as either absolutely true or false. When we come into the domain of physics we are studying the actual, the real, the tangible.

I desire, then, for a few moments to consider, in a general way, matter and force—the one real, positive, palpable, inert; the other immaterial, etherial, incorporeal, yet dominative over the tactile mass; a law which is ever active in bringing about definite changes in the passive matter; a ghostly, pervading something, which changes a dead, a lifeless, an inanimate mass of chaos into this world of life, and joy, and beauty, and animation.

What is this mysterious influence that we call force? Let us examine it. But in what I shall have to say I desire it ever to be borne in mind that I am speaking solely of physics, and that it has no kind of metaphysical or speculative application whatever. The relation of mind to matter, it is no part of my present plan to endeavor to trace out.

Force can be studied only through its chief resulting phenomenon—motion. We find that matter and force are infinitely opposed. Matter seeks eternal rest, force, perpetual motion. They mutually react upon each other, matter being by force constantly changed in its characteristics, force by matter continually varied in mode of manifestation. They are thus mutually interpendent, co-existent, eternal parts of one stupendous whole. Interpendent, because one cannot form a portion of this universe without the other: co-existent, because each pervades the other, and eternal, because both can die only together.

It has long been an axiom in physics that matter is imperishable. The same train of reasoning which proves this, establishes also the fact that force is indestructible. If an atom can change its condition only through the exertion of force, if its form only can be altered, the bonds, which unite it to certain other atoms only be liberated to enable it to form new unions with yet other atoms, and if not one of these particles can be lost or annihilated, then can force only, by the reaction of matter, be induced to change its mode of manifestations or its direction. If not an atom has ever been destroyed since matter existed, then it necessarily follows that not an influence, not a wave of force, has ever been extinguished since, simultaneously with matter, it first exerted sway. We may, through the action of force, alter the condition of matter; we may, through the reaction of matter, change the character of force; but both are unlike indestructible and eternal. Matter is all one, under whatsoever form it may exist. Force is a unit, however it may manifest itself. These are predicates, truisms, self-evident, self-proved, fixed laws of physical science. This doctrine is not new, for as long ago as 1845, Faraday declared that he held the opinion that the various forms under which the forces of matter are made manifest have one common origin, and are so mutually interpendent that they are convertible one into another, and possess equivalents of power in their action. To my apprehension, matter and force are as intimately connected as are what Faraday calls the different manifestations of force with each other. We cannot conceive of matter except as it be subject to force. We cannot imagine power as distinct from the matter upon which it acts. They are essentially co-existent, cœval, synchronous.

Force may act upon matter in different ways, and the result may be motion of a mass, or of atoms. The changes consequent upon this action may be those of external form —morphological—or of internal structure—molecular. According to the peculiar manifestation of it we have been accustomed to call it Heat, Light, Electricity, or Chemical affinity; but in whatever mode it becomes sensible to our preceptions there is one definition which will always describe it, one expression which always characterizes it. It is essentially matter in motion. Heat was formerly regarded as a subtle substance, with unknown, tangible qualities, and its specific name was *Caloric*. It was, with Light and Electricity, classed as an inponderable fluid, because it was conceived that no other hypothesis would account for the phenomena which it exhibited. Light, it was supposed, consisted of minute characteristic particles which proceeded from the sun, or from any luminous body. Electricity also was regarded as an invisible entity of some kind, possessed of peculiar qualities. It was known rather by its physical manifestations than from any knowledge of its character, but the general opinion held it to be an extremely tenuous matter, which, while pervading most substances, could yet be bottled up, confined, or dissipated at will. It was usually spoken of as the electric *fluid*, and in my early school days I was taught that there were two kinds of electricity—a positive and a negative—which were always seeking to neutralize each other. Further study and investigation made manifest the absurdities of these crude theories, and a new hypothesis was invented—that all these supposed entithes, these actual, positive existences of some kind of matter called light, heat, etc., really acted through a specific media—a fluid which pervaded all matter and all space, an invisible, intangible ether, which, once put in motion by the action of light, heat or electricity, had sufficient power to produce all the violent phenomena which were supposed to be the effects of these agents, and this hypothesis is held by most people to-day.

Count Rumford disposed of the material theory by immersing two iron or steel bodies in cold water, and then, by the friction or attrition of the one upon the other, gradually raising the water to the boiling point. This was the initial attempt at removing the consideration of the study of force from the domain of metaphysics to that of physics. Prof. Grove first publicly announced the modern theory that the so-called imponderables—light, heat, electricity, etc.—are peculiar states of ordinary matter; that they are resolvable into motion, and that they are, in fact, all very closely connected, and the new doctrine was denominated the Correlation of Physical Forces. The theory was taken up by others, the real nature of the so-called forces was studied, and the further proposition was enunciated that they are all mutually convertible into each other. It was necessary in the consideration of these forces to study them in their manifestations, to compare them with other physical phenomena, and note their resemblances or their discrepancies. It has been determined that most, if not all the forces, progress by means of an undulating or wavelike motion, unlike the advance of the concentric waves made by casting a stone into a smooth body of water. This hypothesis is firmly established as regards sound, not only by actual measurement of the vibrations of resonant bodies, but by the very structure of our own auditory apparatus.

The vibrations produced by light have not only been demonstrated, but accurately measured. And not only this, but it is very clearly shown that the different colors of the solar spectrum are produced by a definite number of vibrations upon the retina of the eye. Further, the very number of these wave-like beatings have been ascertained and counted. The most delicate, but at the same time the most determining experiments have been conducted, and these demonstrate that to produce the color at one end of the solar spectrum, red, 480,000,000,000,000 of these vibrations must impinge upon the retina in each second; while to produce violet, the color at the other extreme of the spectrum, the number of vibrations per second is no less than 728,000,000,000,000.

The same arguments which are applicable to the undulatory theory of the progress of light, are equally pertinent in the consideration of electricity, for its mode of progression has been shown to be nearly allied to that of light.

And now let us for a moment consider the characteristics of some of these forces. Sir Humphrey Davy says that the immediate cause of the phenomena of heat is motion, and the laws of its communication are precisely the same as the laws of the communication of motion. We know that all molecular movement is accompanied by the evolution of heat to a greater or less degree. This is equally true whether it be of the changes incited by what is known as chemical action, or the motion induced within the mass of iron upon the blacksmith's anvil. We also know that the same molecular disturbance generates what is known as electricity, and that both these elements are operative in inducing that change sometimes called chemism. It is equally true that each of these forces is convertible into any of the others. Thus, if we commence with chemical action, we all know how, within the cells of the battery, this action is made manifest in the electrical current, and thus chemical force is converted into electrical force. If, now, this force be generated in sufficient quantities and conducted along a wire of sufficient size for its easy transportation, and if in this "circuit" a piece of small platinum wire of such size as to partially obstruct the "current" be inserted, we all know that the platinum wire soon becomes red hot, and we see an instance of the conversion of electrical force into heat. The galvano-cautery is an instance of this. If, now, the current be increased and the obstruction be entire at one point, the most dazzling radiance is manifest, and here we have an instance of the conversion of electricity into light. The electric light is an illustration of this.

Here, then, commencing with that simple molecular disturbance within the battery, we see the force generated by those movements manifested first as chemical action. This is converted into electricity, the electricity into heat, and the heat into light, and all without the addition to or the subtraction from the original force as first made manifest, of anything whatever. This proves conclusively that whatever name we may give the phenomena exhibited, they are all due to the same cause, have the same origin, are convertible the one into the other, are in fact all the same thing, differing only in the mode of their manifestation and the accompanying phenomena.

The sun is the great source of light and heat for this earth. The so-called rays of the sun may be made manifest to us in many different ways. If, for instance, we take our stand with that body exposed directly over head, its nfluence is chiefly exhibited to us through that which we call heat. But we may interpose between us and the sun crystals of alum, and these will intercept those undulations which are known to us as heat; or, in other words, it will so change the character of these vibrations that the sun's influence is no longer manifest to us as heat, but the heat rays have become light beams. In other words the heat is converted into light. Again, we may interpose another substance and there is neither heat or light in the sun's influence, but its rays now induce those molecular changes which we know as chemical action. Thus the same ray of the sun may be changed and made manifest to us as light, heat, chemism and electricity.

Force, then, is but a mode of motion, and according to the manner in which it is manifest to our senses we call it by the names which I have considered. But force may remain latent for an indefinite time. In my school-boy days, when we considered heat or caloric, we called it either sensible or latent. Further study will teach us that such terms are the result of our lack of understanding of the subject. There may, in one sense, be such a thing as latent force, but heat is only a method of the manifestation of force. The sun, as I have said, is the origin of all force, because within its body certain changes were originally organized and put in motion, whether by Omnipotent Power, as our system of theology teaches, or by inherent qualities, as materialists claim, matters not in this connection. These molecular changes are the origin of the unit force. The effect of these changes is eternal, imperishable, indestructible. But they are necessarily incessant. They may be stored up, imprisioned, only to be liberated through the transferrence of some other influence, as thus: In the early carboniferous ages, the force liberated through the changes going on within the sphere of the sun being manifested upon the earth as light, heat and chemism, induced certain molecular changes here, which resulted in the new combination of the elements of matter then existent, and the consequence was an extraordinary organic growth, and the formation of the carboniferous forests. The continued force which has its initial point in the sun, still active, but modified by previous changes of the same matter, and by self-limiting, environing circumstances, finally resulted in those immense carbon deposits which to-day form our coal fields. The coal which burns in my grate is but the imprisioned force which was originally derived from the sun, and which came in form of light, heat, electrical and chemical changes. For the proof of this we have but to subject it to favorable influences and there will be returned to nature the same identical light, heat, chemical and electrical forces which so long ago lay inactive, dormant, latent, imprisoned within the coal bed. It is capable of demonstration that the amount returned is the exact amount so long ago received from the sun. But the light, heat, etc., or in other words the force liberated in my grate, is not lost or wasted, but is absorbed, appropriated, perhaps imprisoned, within other masses of matter, to be in turn again yielded up, and again utilized. Or, it may be, the force so eliminated from the coal is at once made manifest in some other mode of motion, and thus transmitted on, and on, now exhibited as heat, now as electricity, and again as light or chemical affinity.

I might enumerate innumerable instances wherein one manifestation of the unit force is changed into another. I might speak of the heat, the light, and the electricity, which in various ways accompany or are the result of chemical action.

I might show that electricity, and light, and chemical action, are ever attendant upon the development of heat, and that heat and light, and electricity, are the accompaniments of the development of chemical action; but it is all summed up in the declaration that all movements of matter, in whatsoever way they may be brought about, are producers of the unit force in some one or more of the methods of its manifestation. I cannot open or shut my jackknife without the evolution of heat and electricity in greater or less degree. If I bring the blade of my knife in quick, sharp contact with another substance sufficiently hard and brittle, heat and light are made evident to the senses, as in the use of the flint and steel.

The evolution of force and its methods of manifestation, are controlled by definite laws which are as yet in a great degree unknown to us. Prof. Grove says, that the law or rule as to the production of heat or electricity from friction or percussion is, that where the mutually inspiring bodies are homogeneous, heat is the consequence; but where they are heterogeneous, electricity is evolved, although either is in a greater or less degree the constant accompaniment of the evolution of the other. In fact, it is true that the production of one force or mode of motion is, as a rule, accompanied with more or less of the others. The beautiful photographic process, which is but the conversion into the molecular motion commonly known as chemical action, is accompanied with the evolution of heat and electricity, though in quantity not appreciable to anything but the most delicate apparatus. If I bend a poker across a chair back, the molecular disturbance of the iron, if it be measured by thermometers and electrometers of sufficient delicacy, will distinctly show an alteration in temperature and electrical condition; and this is true of every change in the relative relation of the atoms which go to make up matter.

Matter is composed mainly of four different elements: oxygen, hydrogen, nitrogen and carbon. Of these four, three are gaseous, and their atoms move freely, and with little friction. Many of the compounds of these elements are what is called allotropic or isomeric—that is, two bodies are composed of exactly the same number of atoms of each element, and yet they are totally unlike, because the relation of the atoms is not the same. Thus the oils of turpentine, lemon and juniper, are chemically the same, yet physically different. So that it is seen that very slight atomic or molecular changes produce wide divergencies in the character of compounds. Then, too, there is abundant opportunity for such changes to be brought about by a very slight exertion of force. The compounds of nitrogen (and this includes all the so called albuminoids) are very unstable, and are ever seeking for some more permanent union. The exhibition of the slightest force is sufficient to induce a disruption of imprisoned chemical affinities which may result in wide changes.

Again, compound matter exists in a number of forms, as gaseous, liquid, and solid. Of these the first two are easily impressed, and molecular changes are constantly going on. Solid matter exists in two different states—the colloid and the crystalloid. Of these the first is unstable and exceedingly mutable. So that of all the forms in which matter exists there is but one in which it is not easily changed and made to assume new molecular conditions. We have shown that every molecular disturbance, however induced, is followed by the evolution or the transferrence of some one or more of the various manifestations of force. Given then, that most forms of matter readily undergo molecular and other changes, and that the manifestations of existent force, such as light, heat, chemism, etc., are constantly active, and that such action can by possibility only result in still another transferrence of force, it may readily be seen how unceasing must be the phenomena presented by all these mutations and mutually induced changes. Every wave of force exerted at the initial period of this universe has been, since that time, and will ever be existent, and either constantly, actively excited, or passively imprisioned by superior force. Matter is, under the action of force constantly being disintegrated, and its constituent particles built anew into fresh forms. And so this tearing down and redistribution of matter is, under the dominion of force, constantly going on. Every organized being, whether animal or vegetable, has its period of molecular aggregation, of growth and so-called nutrition, of active, progressive changes, and then the same forces which have resulted in the combination of the molecules which make up its substance, are again active in those yet further molecular changes which bring about its morphological destruction. I say the same forces which brought together the molecules which composed this body of mine, will in time insure their separation, and thus bring about the disintegration of solid and fluid tissues, and return them again to the common stock of matter, while the energies which brought about these definite changes, through the reaction of the matter thus metamorphosed, will in time be transformed into other forces, and itself returned again to the parent or unit force whence it was segregated, and thus will all that which goes to make up this Ego, this individual I, be returned again to that great source from which it emanated.

If this dictum of the unity and the conditions of forces be admitted as true in its application to the various forces of which we have been speaking, are we not justified in assuming that the law is general throughout the material universe? And whether we study this unity as exhibited in the macro-cosmos, or in the micro-cosmos—in the revolutions of solar systems, or that affinity which binds together two atoms—in those early convulsions which resulted in the upheaval of continents, or the change which cumilates in the growth of a blade of grass—in the devasting earthquake, or the fall of a leaf in autumn—in the wheeling in infinite space of a planet, or the infinitesimal vibrations of a ray of light—in the action of volcanic fires, or the molecular changes within the single battery cell, we shall see that in any extreme it unfailingly exhibits the same characteristics. It is the exhibition of the same force which results in the molecular aggregation called man, and that of the lowest organic life. Within the two organizations constant definite changes are going on that differ but in degree. The results of those changes in the two are precisely alike in fact. Life, vitality, in the one is, in a physical sense, precisely what it is in the other, except that in the lower it is simple and all the processes are elementary, in the higher it is complex and not readily comprehended.

And now, having considered the law of the correlation of forces in its application to the lower forms of matter, shall we stop when we are just upon the threshold of the secret places of nature? We have shown that in inorganic life the law prevails and answers all the phenomena there exhibited. Shall we admit that the harmonies of nature become discords when they are played upon the strings of a more perfect instrument? As we rise in the scale of existence, shall we conclude that where, before, all was beauty, and harmony, and exactness, now all becomes discord, and falsehood, and incongruity? Shall we admit that the laws which are universal in the lower objects, are suspended when we arrive at the point where they are most needed to make things congruous? The world has long accepted as a fact the belief that man is a law unto himself, and that his physical being is not subject to the rules which govern all the rest of creation. The life of one of the lower animals was thought to be one thing, that of man to partake of a very different essence. That the vitality of the shrub which grows by the wayside had no kind of resemblance to the flower which blooms in our gardens. Let us look into this thing.

My subject, as announced, is nervous force. Perhaps many of you have wondered if I were to pay it the respect of a passing glance, and if so, what my long prelude meant. It was necessary that we first establish and make clear to your comprehension the doctrine of the correlation of forces, before we attempted to apply it to other and higher uses.

We have seen that the light and heat of the sun, under favoring conditions, have developed or been transformed into other forces. We have examined those forces, and have found them a unit in their origin, though diverse in their mode of manifestation. We have seen that light may be changed into heat, heat into electricity, and that into chemical affinity. That all these so-called forces are mutually interchangeable, alike, identical. That each is the result of certain molecular changes, themselves induced by manifestations of other varieties of the unit force. We know that all organic bodies, whether of low or high degree, are composed of the same atoms that unite to form other matter, and, therefore, they must be amenable to the same laws.

There are certain phenomena connected with living matter called vital phenomena. Under the old hypothesis that there were many kinds of force, and that each was an entity, acting in an independent manner upon such matter as was subject to its influence, it was easy to suppose that nervous force was a something distinct and by itself, and that it was not subject to the laws which governed other forces. When it was believed that magnetic attraction was a pervading something which established a kind of affection between certain substances, and aversion toward others, and that this attraction was a thing by itself, dominated only by its own laws, and owing no allegiance to the principles which governed the relations of other matter, then it was easy to imagine that nervous force was a principle alike distinct, separate, and removed from all other dominant forces. In that early day there was no harmony in nature, but a continued clashing and discord among mutually contending forces. Let us now suppose that nervous impulse is but another mode of manifestation of the unit parent force, and how quickly all becomes harmony and beauty.

The lapse of time admonishes me that I cannot pursue this enquiry with all the minuteness with which I endeavored to examine the physical forces, but that all force is identical, interchangeable and the same, seems to me plain from a number of reasons In the first place it is derived from the same source. The same molecular changes and mutations which in the battery cell result in the evolution, or more strictly speaking the segregation of electrical force. We are constantly supplying the elements of this nervous battery, in the food which we take, and these molecular changes which we denominate digestion and assimilation, result as such changes ever do, and must result, in the elimination of a force which, in this method of manifestation, we call nervous force.

If an animal be deprived of every kind of food except fats, it finally dies of inanition, though there is no apparent emaciation. The changes incident and necessary to nutrition cannot be carried on in the absence of necessary elements. So the molecular changes of the digestive process having partially ceased, there is a consequent diminution in the evolution of nervous force, which finally results in complete functional stasis, or death.

Again, that nervous force is identical with the other forces is manifest from the fact that in many of its phenomena it is the same. As the light and heat are modified by other forces, as well as by the circumstances under which they are made manifest, so nervous force is dominated by the environments which surround its elimination and exhibition. The methods in which the changes which result in light and heat progress, the elements taking part in such changes, all have an influence upon the characteristic of the force so generated. This is also true of nervous force. When the molecular changes going on within the body in which is generated nervous force are most active, the force generated is great. When these changes cease, nervous force is no longer generated, and the body is dead. When the products of these changes are for any reason transformed into heat, as in certain pathological conditions like fever and inflammations, nervous force is decreased. If the body be subjected to intense cold, the transformation of these changes into force is retarded, and not only is the temperature of the body reduced, but nervous force is adminished, and the organs which are controlled and regulated by it become torpid. Certain drugs have the power entirely to suspend these transferrences of force, or to modify them greatly. So in the generation of other forces through the chemical or changes which induce them, the elimination or action may be modified or suspended by the introduction of interfering matter.

Nervous force may be changed into other forces, and on the other hand, light, heat and electricity may be transformed into nervous force. It is not sufficient that the tadpole be furnished with the necessary food and heat for its development into a frog. Unless light be given him he remains in his tadpole state. If heat be not supplied to the freezing animal there will be no nervous force, and how familar is every physician with the fact that when nervous force seems exhausted, the mere application of heat supplies the needed nervous impulse; how else than by a transferrence of the force? If I apply the poles of a powerful battery to the nerves of an animal in which the evolution of nervous force is quite suspended, all the effects of that force are manifested. The heart can be made to beat, and any special muscle to act as in life, for a limited time; how else than by the transferrence of this mode of manifestation of the unit force? Electricity seems more nearly correlated to nervous force, than is any other mode of motion. Indeed, in some animals they seem interchangeable at will. Thus the gymnotus, or electrical ell, by the possession of a more than usually complicated nervous apparatus, can give electrical shocks of a considerable power at volition.

The fire flies and glow-worms are also provided with special organs by means of which they can at will emit light, as the gymnotus does electricity; that is, nervous force is transmitted into light. In all such animals, when nervous force is exhausted, when they are tired out by continued irritation or excitement, this power to emit light or electricity is gone, and it returns only when the nervous impulse is again perfect.

The laws which govern the manifestation of nerve impulse are less understood than those dominating the other forces. The force itself seems, like an algebraic expression, to be raised to a higher power, but that it therefore differs from the others does not follow. When in the light of the theory of the correlation of forces it is intelligently studied, we may hope that its phenomena will be better understood, and its conservation become a wrought ou problem. We have learned how electricity may be stored up, imprisoned against a time of need. Why should we not discover the same thing concerning its nearly related nervous force? When the battery ceases to work we know how, within certain limits, to remedy the defect. What hinders our learning the same thing of the nervous system? Many men have striven to gain this knowledge, but not, so far as I know, in the light of the latest revelations of science. Nervous force has been regarded as electricity once was: as an entity, an entirety; as something distinct from other forces. It is time that men began its investiga-on from another standpoint.

In the first pages of this hastily written paper I said that the progression of force is, so far as we know, by undulations, and onward, wave-like motions. This is by experiment demonstrated to be true of nervous force. Even the rate of this advance has been determinately measured, and found to be in the motor nerves about 140 feet per second; so that we see in its mode of progression it obeys the law governing other forces.

Of the manner in which nerve force is eliminated we know little, but that it is in some way through the nerve-centres we are convinced. Experiment has proved this and at the same time established the fact of its close corre-lation to the other forces.

When the nerve centres are destroyed or paralyzed, not only is the production of nerve force stopped, but the body quickly cools. Upon sending a current of electricity along the course of the nerves, the bodily heat or temperature rises, so closely are these forces connected. If an organic body be deprived of light, not only is nervous force dimin ished, but the temperature is lowered.

We have all known persons whose hair during conditions of nervous excitement would stand on end, and from whom at such times could be drawn distinct electric shocks. I

know a man who, by inducing a restless, agitated nervous state in favorable atmospheric condition, can light a gas jet by simply holding his finger tip to the burner. These states are always succeeded by nervous depression, undoubtedly due to a loss of nervous power, through its transmutations into electrical force.

That nervous force is very closely correlated with electrical force.

That nervous force is very closely correlated with electrical force is again proved by the fact that all persons of highly wrought nervous organization suffer extremely during electrical disturbances. So-called magnetic storms induce a condition of great nervous exaltation in many people. Nervously anæmic people derive strength from a gentle electric current, because of its conversion into nervous power. People who suffer from nervous irritability find an exacerbation into electricity. That is, when the lesion is of the nerve centres, the generators of nerve force, electricity is beneficial; when in the conducting nerve filaments, it is aggravative, for obvious reasons.

There are many other points and arguments which I should be glad to present, but this paper is already too long, and I must leave the consideration of the subject. I desired to say something concerning a kind of nervous ebb and flow in certain of the vegetable kingdom—to speak of the stinging nettles, and of certain jelly-fishes which without a discoverable nervous system, yet give distinct shocks through some occult means—to speak further of the inordinate waste of nervous force in certain states of excitement or passion—to say something about the anotomy of the nervous system, and to examine a little the phenomena of excessive nervous irritability I am even leaving almost untouched one great division of my subject—nervous lesions. I can only plead the vastness of the subject, and the impossibility of doing more than to make a brief presentation of it within the limits of a paper like this.

The importance of a more careful study of the physiology of nervous forces is apparent when we remember that the type of American diseases is to-day distinctly nervous, and that from year to year it is growing more so. Reflecting men in the medical profession have begun to recognize that we are making little progress in learning to combat these ills, and are seriously looking about for the reason. Some of the most profound thinkers in medicine have turned their attention almost exclusively to this field. They have advanced little further than to discover the cause of certain troubles, and lament the inability of the profession to grapple with and overcome the difficulty. Books have been written which have stirred medical men up to a recognition of the importance of this subject, without convincing their authors that they themselves fully comprehend the matter. It it not time that enquiry took another direction? If any one has studied the subject from the vantage ground of the correlation of nervous impulse with the other forces, I am not aware of it; but I hope that this may be a door which shall enable some one to enter upon a field that will give richer returns than any have yet yielded.

